# Biological characteristics of molecular subtypes of ulcerative colitis characterized by ferroptosis and neutrophil infiltration

**DOI:** 10.1038/s41598-024-60137-z

**Published:** 2024-04-25

**Authors:** Shaopeng Sun, Yuqing Mao, Sihua Le, Mingxu Zheng, Menglin Li, Yifei Chen, Jiajia Chen, Yihong Fan, Bin Lv

**Affiliations:** 1 Department of Gastroenterology, The First Affiliated Hospital of Zhejiang Chinese Medical University (Zhejiang Provincial Hospital of Traditional Chinese Medicine), No. 54, Youdian Road, Shangcheng District, Hangzhou, 310003 Zhejiang China; 2 The First School of Clinical Medicine, Zhejiang Chinese Medical University, Hangzhou, China; 3 Department of Nursing, The First Affiliated Hospital of Zhejiang Chinese Medical University (Zhejiang Provincial Hospital of Traditional Chinese Medicine), Hangzhou, China

**Keywords:** Ulcerative colitis, Ferroptosis, Neutrophil, Immune infiltration, Treatment response, Computational biology and bioinformatics, Immunology, Gastroenterology

## Abstract

Clinical ulcerative colitis (UC) is a heterogeneous condition. Moreover, medical interventions are nonspecific, and thus, treatment responses are inconsistent. The aim of this study was to explore the molecular subtypes and biological characteristics of UC based on ferroptosis and neutrophil gene sets. Multiple intestinal mucosa gene expression profiles of UC patients in the Gene Expression Omnibus (GEO) database were downloaded. Unsupervised clustering methods were used to identify potential molecular subtypes based on ferroptosis and neutrophil gene sets. Multiple immune infiltration algorithms were used to evaluate the biological characteristics of the molecular subtypes. Machine learning identifies hub genes for molecular subtypes and analyses their diagnostic efficacy for UC and predictive performance for drug therapy. The relevant conclusions were verified by clinical samples and animal experiments. Four molecular subtypes were identified according to the ferroptosis and neutrophil gene sets: neutrophil, ferroptosis, mixed and quiescent. The subtypes have different biological characteristics and immune infiltration levels. Multiple machine learning methods jointly identified four hub genes (*FTH1, AQP9, STEAP3 and STEAP4*). Receiver operating characteristic (ROC) curve analysis revealed that the four hub genes could be used as diagnostic markers for UC. The clinical response profile data of infliximab treatment patients showed that *AQP9* and *STEPA4* were reliable predictors of infliximab treatment response. In human samples the AQP9 and STEAP4 protein were shown to be increased in UC intestinal samples. In animal experiments, the ferroptosis and neutrophil phenotype were confirmed. Dual analysis of ferroptosis and neutrophil gene expression revealed four subgroups of UC patients. The molecular subtype-associated hub genes can be used as diagnostic markers for UC and predict infliximab treatment response.

## Introduction

Ulcerative colitis (UC) is an inflammatory bowel disease characterized by alternating periods of active and remission states. UC seriously affects the life of patients and leads to long-term burdensome complications^1^. Over the past two decades, UC has emerged as a global health challenge. Epidemiological data show that the incidence of this disease has plateaued in high-income countries but continues to increase in low- and middle-income countries^2^. The emergence and development of biological agents, such as antitumour necrosis factor (TNF), have led to more options for treatment, but patients still face problems such as a loss of response. The emergence of new therapies allows consideration of more stringent treatment goals, including clinical and endoscopic remission. Currently, histological remission is proposed as the latest therapeutic goal^3^.

The pathogenesis of UC is complex and incompletely understood. It is generally accepted that disruption of the intestinal barrier triggers the initial pathological phase. Intestinal epithelial cells (IECs) constitute the first line of defence of the intestinal barrier, and their abnormal death leads to increased mucosal permeability, pathogen invasion, intestinal immune imbalance and other problems^4^. Ferroptosis is a type of cell death caused by the excessive accumulation of lipid peroxides due to the disruption of intracellular metabolic pathways and is closely related to intracellular iron metabolism and lipid homeostasis^5^. Recently, several studies have demonstrated ferroptosis in IECs in animal models^6–8^. Saving IECs from abnormal death, such as apoptosis and ferroptosis, is a protective strategy of the intestinal barrier^9^. Our previous studies revealed ferroptosis in mucosal samples from humans and colitis animals, and inhibiting ferroptosis improved symptoms and prolonged survival in mice with colitis^8^. The process of ferroptosis is regulated by various molecular mechanisms, such as the regulation of iron, lipid metabolism and the control of reactive oxygen species (ROS). The sensitivity of cells to ferroptosis is the key factor restricting the application of the ferroptosis pathway^10^. Therefore, identifying UC patients who are sensitive to ferroptosis is a prerequisite for effective therapy.

Neutrophils are considered complex innate immune cells that play a key role in maintaining intestinal mucosal homeostasis^11^. Neutrophils play dual roles in regulating the pathogenesis and repair of IBD^12^. They play a substantial proinflammatory role in the acute phase of pathogen infection and intestinal inflammation and recruit other immune cells. They also limit the invasion of foreign microorganisms and promote mucosal repair^13^. Recent studies have shown that neutrophils also have different phenotypes. Specific subsets of neutrophils are thought to be involved in different physiological functions, including angiogenesis, immunosuppression, and/or epithelial protection^14–16^. Neutrophils exhibit multiple functions and are considered interesting targets for interventional therapy^17^.

In this study, we explored the molecular subtypes and biological characteristics of UC based on ferroptosis and neutrophil gene sets. Furthermore, machine learning methods were used to identify central genes associated with molecular subtypes, explore the diagnostic efficacy of hub genes and predict the therapeutic response to biologics.

## Methods

### Patients and samples

We obtained the intestinal mucosal expression profile data of UC patients and healthy individuals from four GEO datasets. The GSE38713 dataset contained 43 biopsies: 13 from healthy control individuals and 30 from patients with UC^18^. The GSE87473 dataset included colon mucosal biopsies from 21 healthy participants and 106 UC patients^19^. The data compared between baseline UC patients (n = 87) and healthy control individuals (n = 21) and between week 6 (n = 75) and baseline patients prior to treatment were included in the GSE92415 cohort^20^. The intestinal mucosal transcriptome data and clinical response characteristics of 24 UC patients before and 4–6 weeks after the first infliximab (IFX) treatment were recorded in the GSE16879 dataset^21^. The “ComBat” function in the “sva” package was used for batch correction to remove non biological effects.

We also obtained intestinal mucosal samples from active UC patients and healthy volunteers (HCs) at the Endoscopy Center of the First Affiliated Hospital of Zhejiang Chinese Medical University. According to our previous methods^22^, some of the samples were cut into paraffin sections and then subjected to immunohistochemistry (IHC) analysis. The remaining samples were subjected to Western blot (WB) quantitative analysis. This study was approved by the Ethics Committee of the First School of Clinical Medicine, Zhejiang Chinese Medical University (no. 2022-KL-133-01). Written informed consent was obtained from all participants before their enrolment. All methods were carried out in accordance with relevant guidelines and regulations.

### Animal experiments

Male specific pathogen-free (SPF) C57BL/6 mice of 6–8 weeks of age were obtained from the Shanghai Laboratory Animal Center (SLAC). An animal model of UC was constructed according to our previous method^8^. In brief, the experimental group was given 3% dextran sodium sulfate (DSS)-distilled water for 7 consecutive days, while the control group was given normal distilled water. The body weights of the mice were recorded daily. On day 7, the mice were euthanized. Euthanasia was performed by CO2 asphyxiation. Then, the entire colon was isolated intact, and its length was measured. Haematoxylin and eosin (HE) staining was used to observe the pathological morphology of the intestinal mucosa. Transmission electron microscopy (TEM) was used to observe the mitochondrial morphology of intestinal mucosal cells. Iron tissue and malondialdehyde levels were measured to determine the colon iron concentration and malondialdehyde concentration, respectively. Animal experiments were approved by the Institutional Animal Care and Use Committee of Zhejiang Chinese Medical University (no. IACUC-20220321-04). All methods for animals were performed in accordance with the relevant guidelines and regulations. This study is reported in accordance with ARRIVE guidelines (https://arriveguidelines.org).

### Feature selection and construction of UC subtypes

The neutrophil hallmark gene sets from the study by Xiao et al.^23^ were used. Ferroptosis hallmark gene sets were obtained from the Molecular Signature Database (MsigDB, www.gsea-msigdb.org/gsea/msigbd/). A total of 64 ferroptosis-related genes and 34 neutrophil-related genes were included in the study, and all the genes are recorded in the supplementary document (Supplementary files: S1). The subgroups were identified based on the expression of ferroptosis- and neutrophil-related genes using unsupervised consistent clustering. For each subtyping procedure, samples underwent consensus clustering based on each hallmark gene, followed by semiautomatic subtype assignment based on gene expression patterns. The clustering process was carried out using the R package “ConsensusClusterPlus”^24^.

The median expression level (z-score) of the expression of ferroptosis and neutrophil genes was calculated for each sample and used to assign associations with these two pathways. For example, when the z-score of ferroptosis signature was > 0 and the z-score of neutrophil signature was > 0, we defined the group as “Mixed”, meaning that the patients in this group had high levels of both characteristic genes.

### Assessment of immune infiltration according to molecular subtype

Four immune infiltration algorithms were adopted in this study. A Microenvironment Cell Population-counter (MCPcounter) allows robust quantification of the absolute abundance of eight immune cell populations (B cell, T cell, CD8 + T cell, monocytic cell, myeloid dendritic cell, neutrophils, NK cells and cytotoxic lymphocytes,) and two stromal cell populations (endothelial cells and fibroblasts) in heterogeneous tissues from transcriptomic data^25^. The “McPcounter.estimate” function of the R package “MCPcounter” was used to estimate the immune cell count. Single-sample gene set enrichment analysis (ssGSEA) was performed based on the ssGSEA projection methodology described by Barbie et al.^26^. The relative enrichment of each gene set in that sample was estimated by comparing the gene expression data of each sample with a specific gene set (the immune cell gene set). The ssGSEA was performed using the ssgsea parameter in the “GSVA” R package. QuanTIseq was used to determine the proportions of ten different immune cell types and the fraction of other uncharacterized cells present in the heterogeneous sample by deconvolution^27^. Cell-type identification by estimating relative subsets of RNA transcripts (CIBERSORT) is a computational method for evaluating the relative abundance of different cell types in complex mixed tissue samples^28^. CIBERSORT provides gene expression profiling data for 22 immune cells (including 7 T-cell types, naive and memory B cells, plasma cells, NK cells, and bone marrow subsets). These analyses were completed using the R package “CIBERSORT”.

### Biological characteristics of the molecular subtypes

Weighted correlation network analysis (WGCNA) can be used to identify cocorrelated genes, identify gene sets with potential coregulatory patterns, and divide genes into different modules. In this study, we constructed a weighted gene coexpression network using the R “WGCNA” package^29^ with approximately scale-free properties^30^. The presence of highly cooperative genes was determined by the correlation between the expression values of all these genes. The network modules were generated by topological overlap measurement (TOM)^31^, and coexpressed gene modules were identified using a dynamic hybrid cutting method (bottom-up algorithm)^32^. Eventually, modules with related genes were merged. Correlations between genes and modules were measured by calculating gene significance and module significance. Gene set enrichment analysis (GSEA) was performed using GSEA software^33^. Kyoto Encyclopedia of Genes and Genomes (KEGG) systematically analyses the functions of genes based on known biological processes in cells and the optimal interpretation of gene functions^34^. The “clusterProfiler” package of the R language was used for KEGG enrichment analysis.

### Machine learning screening for diagnostic genes

Transcriptome data from all UC patients (n = 298) and healthy subjects (n = 55) in three GEO datasets (GSE87473, GSE92415, and GSE38713) were used to construct the diagnostic model. The Boruta algorithm is a wrapper feature selection method that compares the importance of the original feature and the randomly generated shadow feature. A feature that is significantly better than a shadow feature is labelled an “important feature”^35^. Least absolute shrinkage and selection operator (LASSO) regression^36^ can reduce the impact of multicollinearity on regression results by reducing the coefficient of the relevant independent variables to 0 through the correlation between them. Support vector machines (SVMs)^37^ are supervised learning algorithms used to solve binary classification problems. This method can find an interface so that all samples are correctly divided into two categories, and the distance between the samples and the interface is maximized. RandomForest^38^ is a classifier that contains multiple decision trees, and the category of its output is determined by the mode of the categories output by the individual trees. The parameter “MeanDecreaseGinimeasures”, based on the Gini index, measures the importance of a gene. Extreme gradient boosting (XGBoost)^39^ is a boosting algorithm that can integrate many weak classifiers to form a strong classifier. In this study, XGBoost was implemented using the “xgboost” package in the R language. The five different machine learning methods described above were used to screen for the best characteristic variables (the best diagnostic genes) to distinguish UC patients from healthy individuals.

Artificial neural networks (ANNs) are mathematical models based on biological neural networks^40^. Gene scores were obtained by analysing the expression levels of the genes and comparing them to the median expression of all the samples. When the gene expression level was upregulated but the representative gene expression level was low, the value was 0; otherwise, the value was assigned to 1^41^. The ANN model was developed by applying the R package “neuralnet”, and the model included five hidden layers. The sum of each input gene score multiplied by the weight of each input gene determines the final output layer.

### Statistical analysis

All analyses were performed with R software (version 4.3.1, https://www.r-project.org). Microarray data were combined and normalized using the R package “limma”^42^. The correlation of normally distributed data was determined by Pearson correlation analysis; otherwise, Spearman correlation analysis was used. The Wilcoxon test was used to compare the significance of factor differences between two groups, and the Kruskal test was used for more than two groups. A two-tailed p value < 0.05 was considered to indicate statistical significance. Benjaminiand Hochberg (BH) method was used for multiple correction. The sensitivity and specificity of the model were evaluated by receiver operating characteristic (ROC) curve analysis using the R package “pROC”. Single, double, triple and quadruple asterisks represent P < 0.05, P < 0.01, P < 0.001 and P < 0.0001, respectively.

### Ethical approval

This study was approved by the Ethics Committee of the First School of Clinical Medicine, Zhejiang Chinese Medical University (no. 2022-KL-133-01). Animal experiments were approved by the Institutional Animal Care and Use Committee of Zhejiang Chinese Medical University (no. IACUC-20220321-04).

## Results

### Dual analysis of ferroptosis and neutrophil gene expression identifies four subgroups of UC

**Figure 1:**
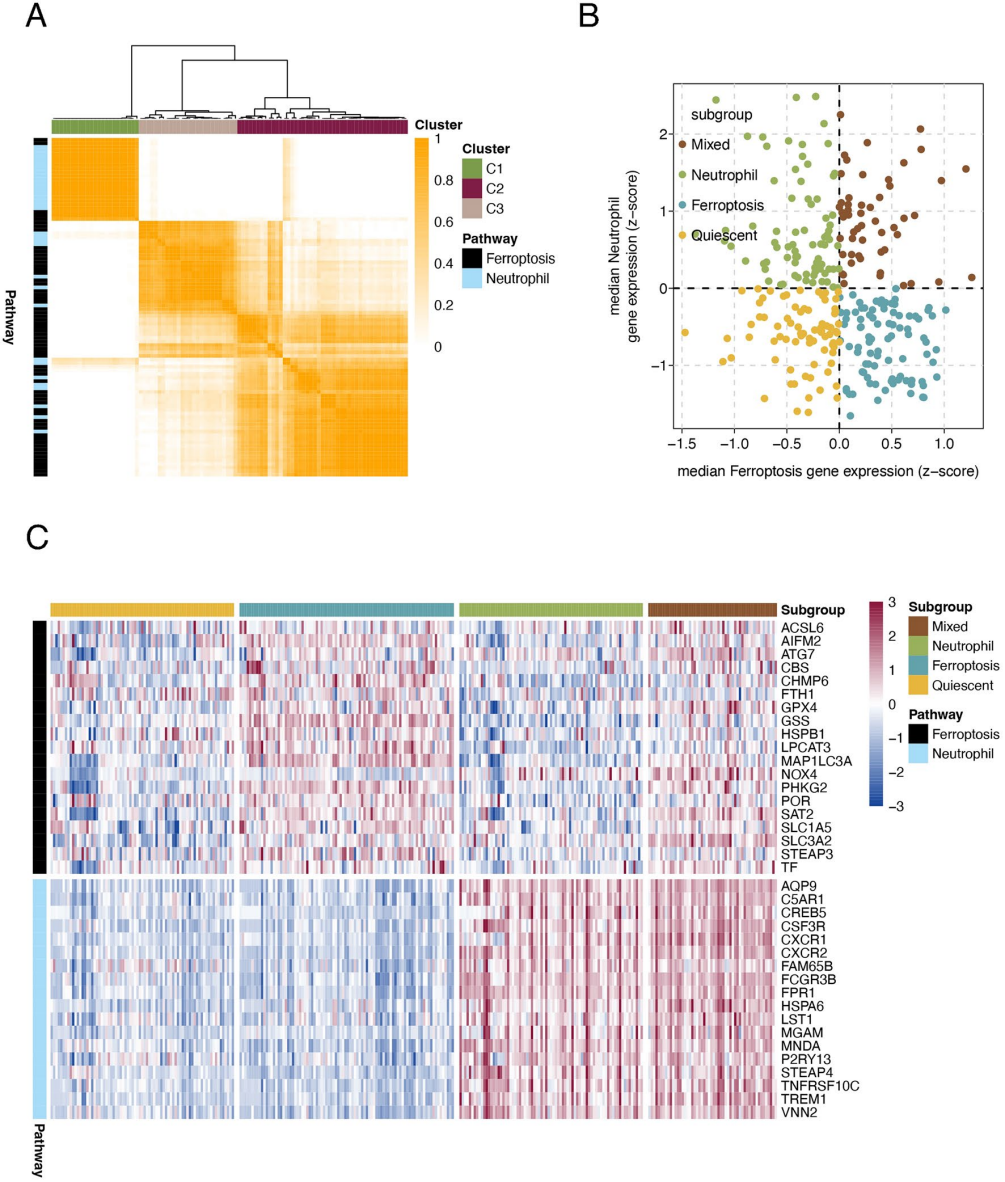
UC was stratified based on the expression of ferroptosis-related and neutrophil-related genes. (**A**) Heatmap depicting the consensus clustering solution (k = 3) for ferroptosis and neutrophil genes in UC samples (n = 298). (**B**) Scatter plot showing the median expression levels of ferroptosis (x-axis) and neutrophil (y-axis) genes in each UC sample. (**C**) Heatmap depicting the expression levels of major subtype-contributing genes across each subgroup.

To stratify UC based on the relative expression levels of ferroptosis- and neutrophil-related genes, we utilized microarray data from the GEO. The complete UC patient mRNA data (n = 298) were obtained from three different GEO datasets (GSE38713, GSE87473 and GSE92415). The ferroptosis (n = 64) and neutrophil (n = 34) gene sets were selected for analysis. To aid in selecting genes coregulated within each pathway that are relevant to UC, we used consensus clustering to identify two groups of robustly coexpressed ferroptosis and neutrophil synthesis pathway genes for UC subtyping (Fig. 1A). The C1 cluster was mainly composed of neutrophil-related genes, while the C2 cluster was mainly composed of iron death-related genes. The median expression levels of coexpressed ferroptosis and neutrophil genes were calculated for each sample and used to assign one of four profiles specifically relevant to these two pathways: quiescent, ferroptotic, neutrophilic and mixed (Fig. 1B, Supplementary files: S2). The ferroptosis phenotype was the most common phenotype (90/298, 30.2%), followed by the neutrophil (77/298, 25.9%) and quiescent (77/298, 25.9%) phenotypes, and the mixed phenotype had the lowest proportion (54/298, 18.1%). The expression levels of ferroptosis- and neutrophil-related genes across the UC subgroups are shown in Fig. 1C. A total of 37 genes (19 ferroptosis genes and 18 neutrophil genes) were defined as major subtype-contributing genes (Supplementary files: S3).

### Molecular subtype patterns regulate the mucosal immune microenvironment in UC

**Figure 2:**
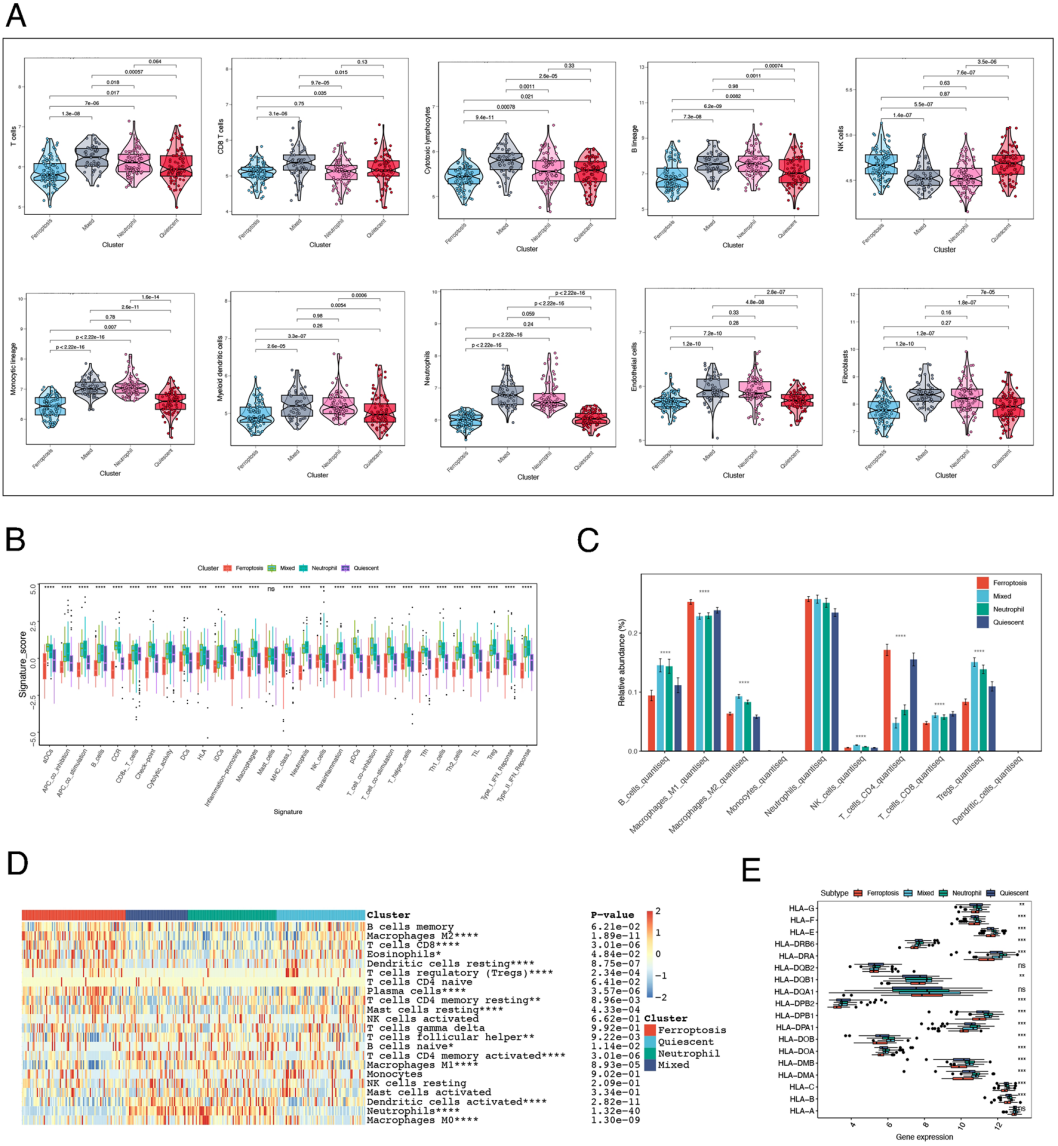
Molecular subtype immune infiltration levels based on multiple algorithms. Immune infiltration was evaluated using the MCPcounter (**A**), ssGSEA (**B**), quanTIseq (**C**) and CIBERSORT (**D**) algorithms. (**E**) The expression levels of HLA-related genes across the four subgroups.

Immune abnormalities are the core mechanism of IBD, so we evaluated the immune microenvironment of the four molecular subtypes by different immune cell algorithms. Here, we used four immune infiltration algorithms. The MCPcounter results showed that the number of NK cells in the ferroptosis group was greater than that in the control group, while the number of other immune cells was lower (Fig. 2A). The results obtained from the ssGSEA also confirmed this finding (Fig. 2B). In addition to neutrophils, a variety of immune cells (T cells, B cells, monocytic cells, and myeloid dendritic cells) and stromal cells (endothelial cells and fibroblasts) maintained high expression levels in the neutrophil and mixed groups (Fig. 2A). ssGSEA, quanTIseq and the CIBERSORT algorithm also produced similar results (Fig. 2B-D).

In addition, we further compared differences in expression levels of HLA-related genes across the four subgroups. Similarly, the ferroptosis group still exhibited a low expression level, followed by the quiescent group, and the neutrophil and mixed groups maintained a high expression level (Fig. 2E). Based on the above analysis, the level of immune infiltration was lower in the ferroptosis group and the quiescent group, while the neutrophil and mixed groups had a greater level of immune infiltration.

### Molecular subtypes present unique biological characteristics

**Figure 3:**
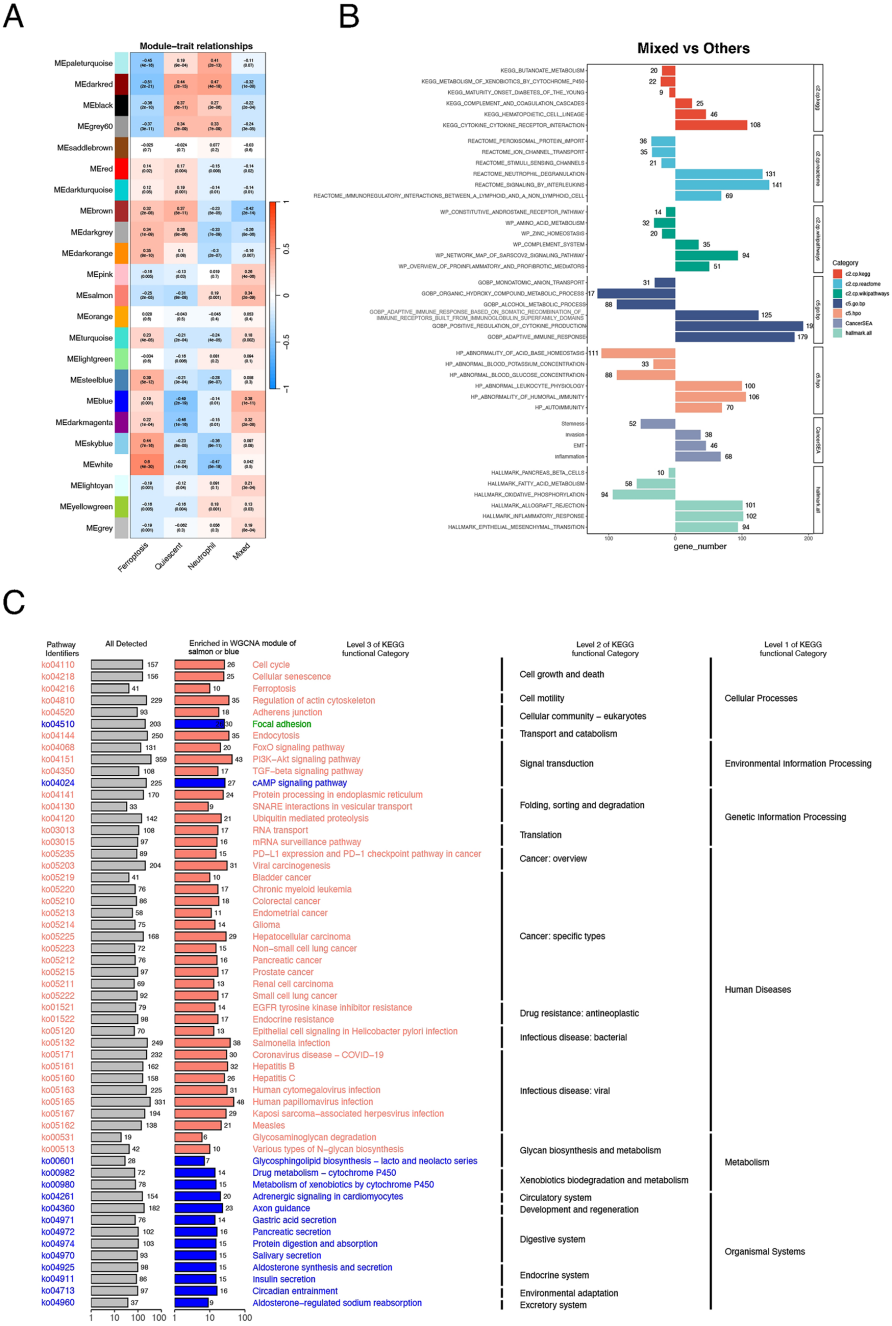
Heterogeneous functional enrichment of molecular subtypes. (**A**) Gene modules significantly associated with molecular subtypes were classified using the WGCNA algorithm. (**B**) GSEA was used to compare the biological characteristics of the mixed group and other groups. **C**, KEGG analysis of gene modules positively correlated with mixed phenotypes.

To further explore the molecular biological characteristics of the four subtypes, WGCNA was first used to find modules of highly correlated genes in the four subtypes. A total of 23 gene modules significantly associated with phenotype were retained (each module was assigned a different colour block, such as MEblack). Correlations between gene modules and molecular subtypes are represented by red (positive correlation) or blue (negative correlation), and the depth of the colour indicates the degree of correlation (Fig. 3A). The four subtypes exhibit significant heterogeneity, suggesting that their respective biological functions differ.

The biological function of the mixed group is the focus of our attention because these cells exhibit high expression of ferroptosis- and neutrophil-related genes. Therefore, we conducted GSEA on the mixed group samples and other samples to identify the unique biological characteristics of the mixed group. The results showed that, compared with those in the other three groups, the genes in the mixed group were significantly correlated with biological pathways such as receptor interaction, interleukin, lymphocyte, cytochrome production, immune, and proinflammatory response pathways (Fig. 3B). The gene modules (MEsalmon and MEblue) enriched in the WGCNA and positively correlated with the mixed phenotype were used for further KEGG analysis. The three-level classification of the enriched biological pathways is shown in Fig. 3C. The mixed group was related mainly to cellular processes, environmental information processing, genetic information processing, human diseases, metabolism and organismal systems. The ferroptosis pathway was also enriched in the categories of cell growth and death.

### Targeting four molecular subtype-associated genes through machine learning

**Figure 4:**
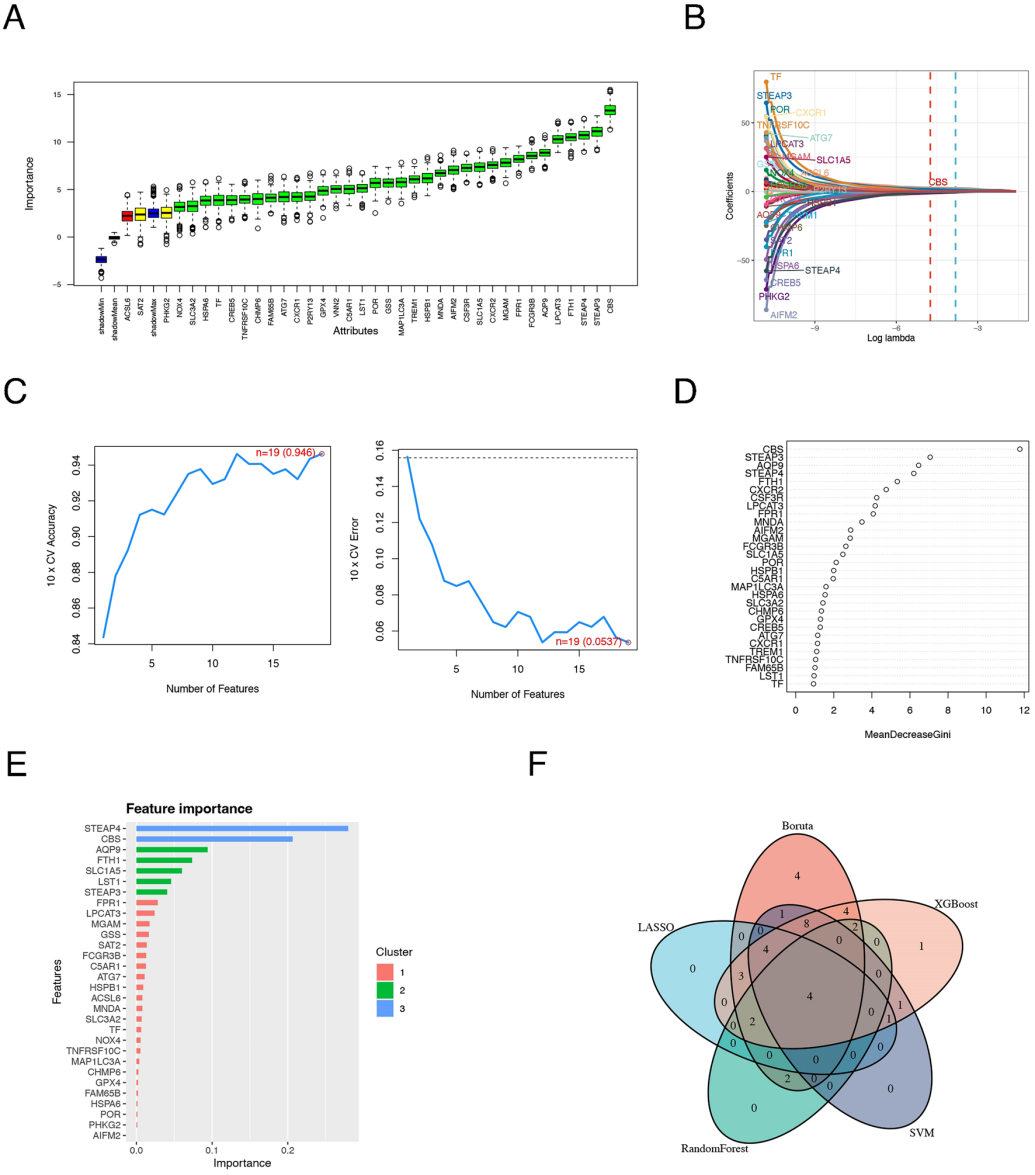
Machine learning to screen for diagnostic genes. Diagnostic gene selection was performed using the Boruta (**A**), LASSO (**B**), SVM (**C**), random forest (**D**) and XGBoost (**E**) models. (**F**) The intersection of the Venn diagram reveals the final diagnostic genes.

A definitive diagnosis is essential for the management of patients with UC, and the discovery of key genes that influence disease progression means early detection, early treatment, and even the development of more effective drugs. We applied five different machine learning methods to the 37 subtype contribution genes (Supplementary files: S3) obtained above to evaluate their diagnostic validity. The Boruta algorithm was used to determine the importance of 37 genes (Fig. 4A). The top six genes were *CBS, STEAP3, STEAP4, FTH1* and *AQP9*. The LASSO algorithm calculates a coefficient for each gene (Fig. 4B), while the binomial deviation under different lambdas helped us retain 14 important genes (Supplementary file S4). The SVM algorithm uses 10 × cross-validation. When the feature vector is 19, the accuracy is highest, and the error rate is lowest (Fig. 4C). RandomForest was used to calculate the “MeanDecreaseGini” for each gene, after which the genes were sorted (Fig. 4D). The XGBoost algorithm was used to score and rank the feature genes according to their importance (Fig. 4E). The hub genes screened by five machine learning methods can be found in Supplementary files S4–S9. Eventually, two ferroptosis marker genes (*FTH1* and *STEAP3*) and two neutrophil marker genes (*AQP9* and *STEAP4*) were screened via a Venn diagram (Fig. 4F). More detailed machine learning results can be found further in the Supplementary files S10.

### Molecular subtype-associated hub genes can be used as diagnostic markers for UC

**Figure 5:**
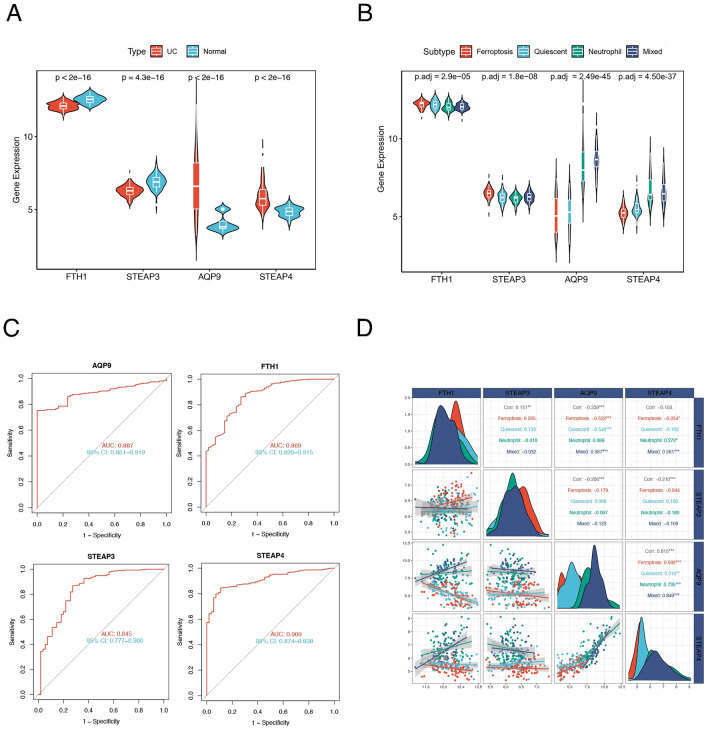
Diagnostic efficacy of the four molecular subtype-associated hub genes. (**A**) Expression levels of 4 hub genes in UC patients and healthy individuals. (**B**) Expression levels of *FTH1*, *STEAP3*, *AQP9* and *STEAP4* in the four subtypes. (**C**) ROC curves of the 4 hub genes. (**D**) Correlations between four hub genes and patient subtypes.

The four hub genes screened by machine learning showed significant differences in expression between UC patients and healthy individuals (Fig. 5A) as well as between the four molecular subtypes (Fig. 5B). The correlations between the four genes and patient subtypes are shown in Fig. 5C. ROC curves of the 4 hub genes revealed that the area under the curve (AUC) values of *AQP9*, *FTH1*, *STEAP3* and *STEAP4* were 0.887, 0.869, 0.845 and 0.909, respectively, indicating the high diagnostic efficiency of these four genes (Fig. 5D).

**Figure 6:**
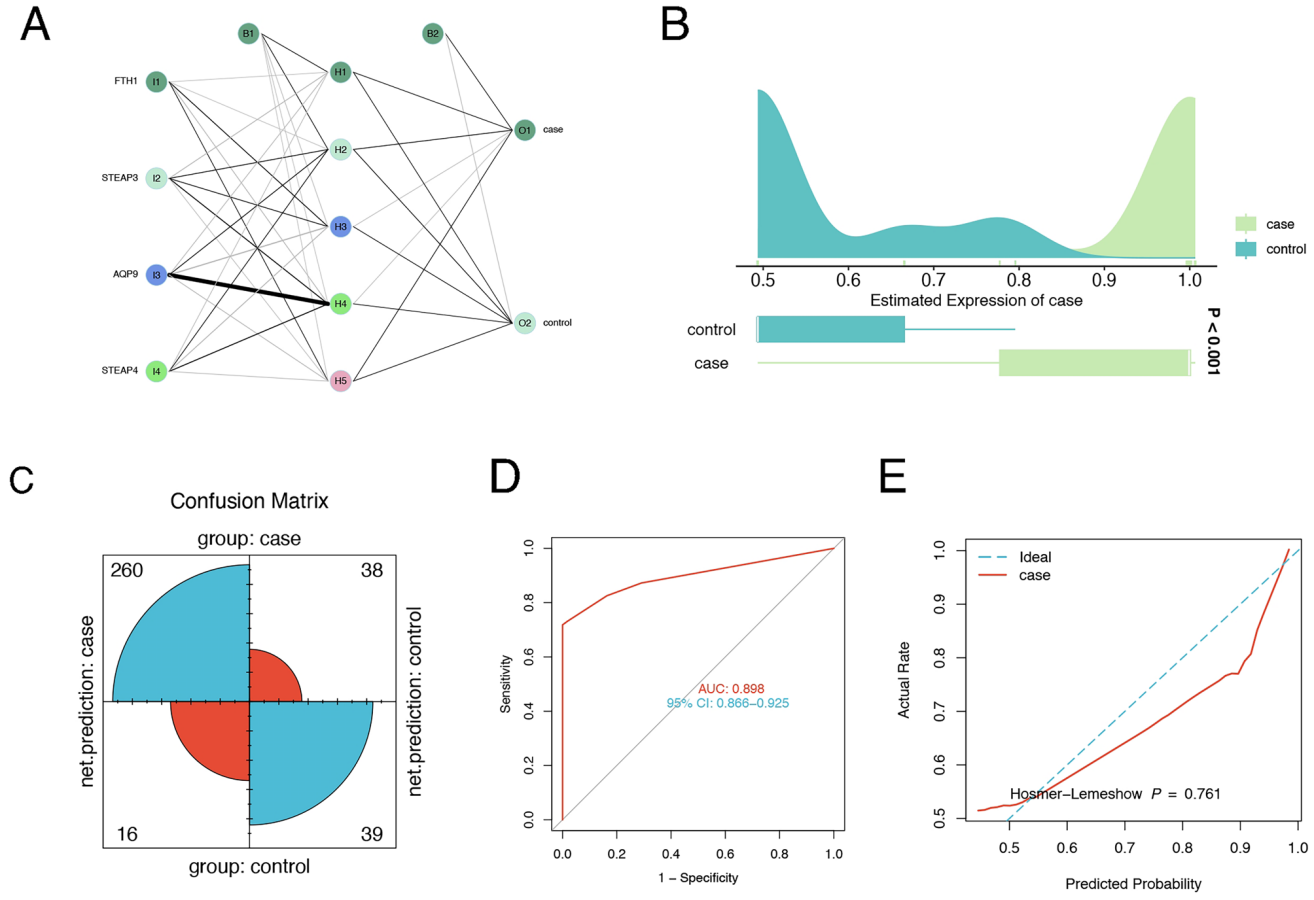
The combined diagnostic efficiency of the hub genes based on an artificial neural network. (**A**) Construction diagram of the neural network. (**B**) The distribution and differences of neural network scores. (**C**) The confusion matrix indicates the accuracy of the prediction. (**D**) ROC curve of the combined diagnosis of the 4 genes. (**E**) Goodness-of-fit tests showed no difference between the neural network and the ideal model (p > 0.05).

To further explore the combined diagnostic ability of the four genes, we constructed an artificial neural network (Fig. 6A, Supplement file S9). Figure 6B shows the distribution and differences of neural network scores. The confusion matrix showed that the accuracy of predicting UC was 87.2% (Fig. 6C). The ROC curve shows the efficiency of the combined diagnostic tool (Fig. 6D). A goodness-of-fit test showed that there was no significant difference (P = 0.761) between the neural network and the ideal model (Fig. 6E).

### Molecular subtype-associated hub genes predict treatment response in UC patients

**Figure 7:**
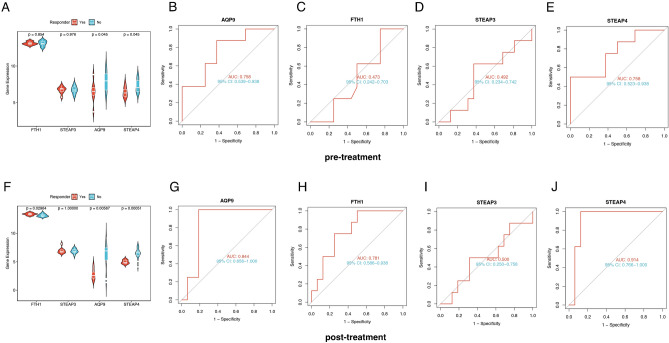
Efficacy of four hub genes in predicting response to infliximab treatment. Differences in the expression of the four hub genes in the responding and nonresponding populations before (**A**) and after (**F**) infliximab treatment. ROC curves of the four hub genes before (**B–E**) and after (**G–J**) infliximab treatment.

IFX treatment improves clinical presentation and induces mucosal healing in UC patients^43^. However, up to 30% of IBD patients with IFX have no clinical benefit and have a primary failure of response. Approximately 20% to 40% of patients achieve a partial response^44^. We therefore used the GSE16879 dataset to further validate the expression levels of four diagnostic genes in responders and nonresponders before and after initial infliximab treatment (Fig. 7A, F). Before IFX treatment, *FTH1* and *STEAP3* did not significantly differ between responders and nonresponders, while AQP8 and *STEAP4* did significantly differ (p < 0.05) (Fig. 7A). The ROC curve also showed that *STEAP4* and *AQP9* (but not *FTH1* or *STEAP3*) were strongly related between responders and nonresponders (Fig. 7B-E). After IFX treatment, there were significant differences in *FTH1*, *AQP9*, and *STEAP4* expression between responders and nonresponders, but *STEAP3* expression was not significantly different (Fig. 7A). ROC curve analysis revealed that *FTH1*, *STEAP4* and *AQP9* had good discriminant efficiency (Fig. 7G-J).

These results suggest that *AQP9* and *STEAP4* can predict patient response to IFX and that IFX treatment may have a significant impact on *FTH1* expression.

### Ferroptosis and neutrophil infiltration occur in human UC intestinal mucosa samples and DSS-induced mouse colon tissues

**Figure 8:**
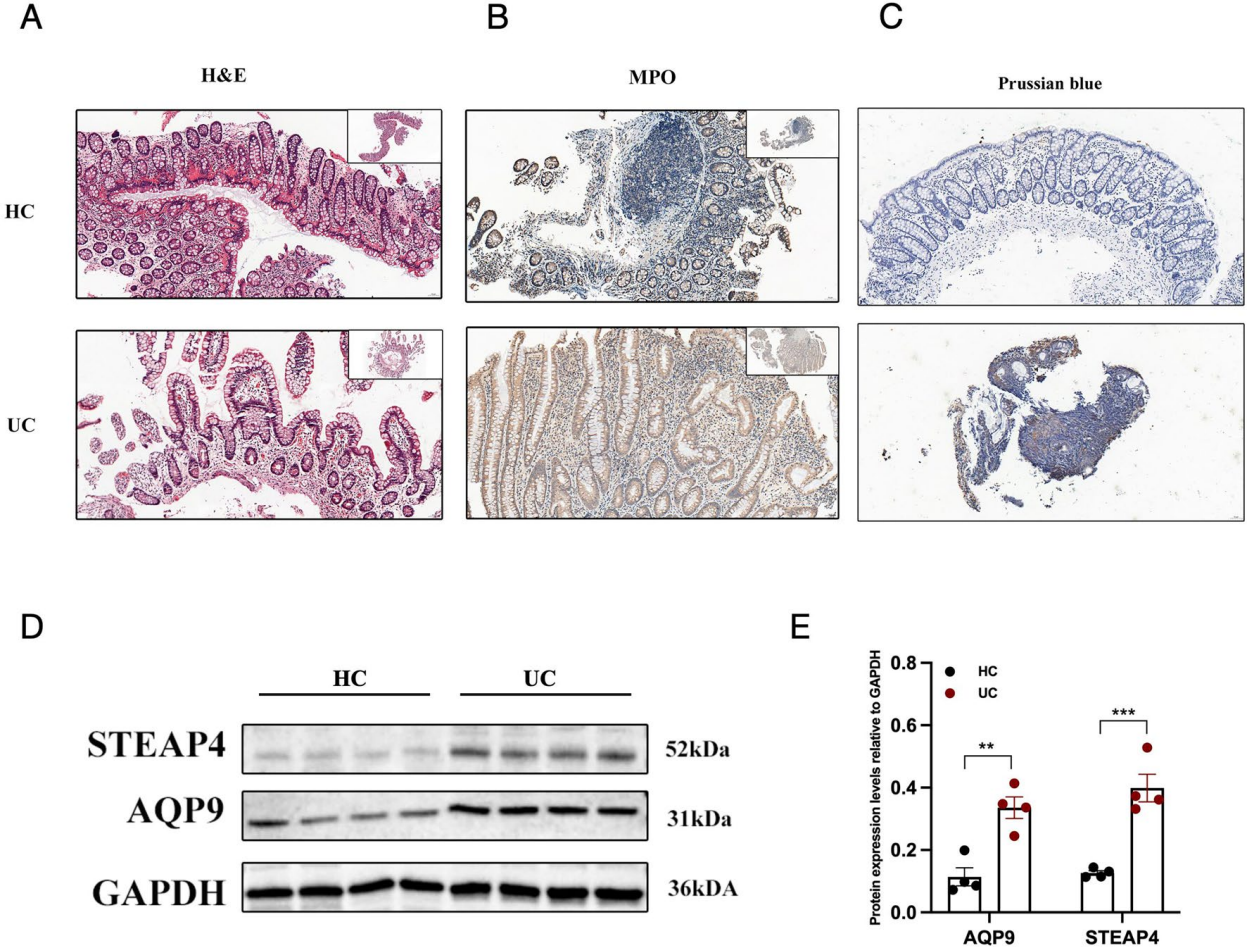
Experimental verification in human UC intestinal mucosa samples. HE (**A**), MPO (**B**) and Prussian blue (**C**) staining of HC and UC samples. (**D**) The protein levels of AQP9 and STEAP4 in HC and UC samples were compared via quantitative WB analysis. Three on the left are from healthy control intestinal mucosa, and three on the right are from intestinal mucosa tissue of UC patients. (**E**) Statistical analysis of the protein levels. Magnification, × 20 (scale 50 μm). **P < 0.01; ***P < 0.001; ****P < 0.0001.

To confirm the above conclusions, we used intestinal mucosal tissues from UC patients and healthy volunteers. HE staining revealed that, compared to those in HC individuals, the glandular arrangement of intestinal mucosal tissue in UC patients was disrupted, with structural damage and infiltration of inflammatory cells (Fig. 8A). MPO staining for neutrophil infiltration (brown: MPO-positive cells) was high in UC patients (Fig. 8B). DAB-enhanced Prussian blue staining revealed trace amounts of iron in tissues, and the intestinal mucosa of UC patients was overloaded with iron (Fig. 8C). In view of the efficacy of *AQP9* and *STEAP4* in diagnosis and drug response prediction, we performed a quantitative analysis of WB data from human samples. Consistent with the above results at the gene level (Fig. 5A), the protein levels of AQP9 and STEAP4 were also significantly upregulated in UC patients compared with HC individuals (Fig. 8D, E). The full-length gels and blots are included in Supplementary Fig. S1.

**Figure 9:**
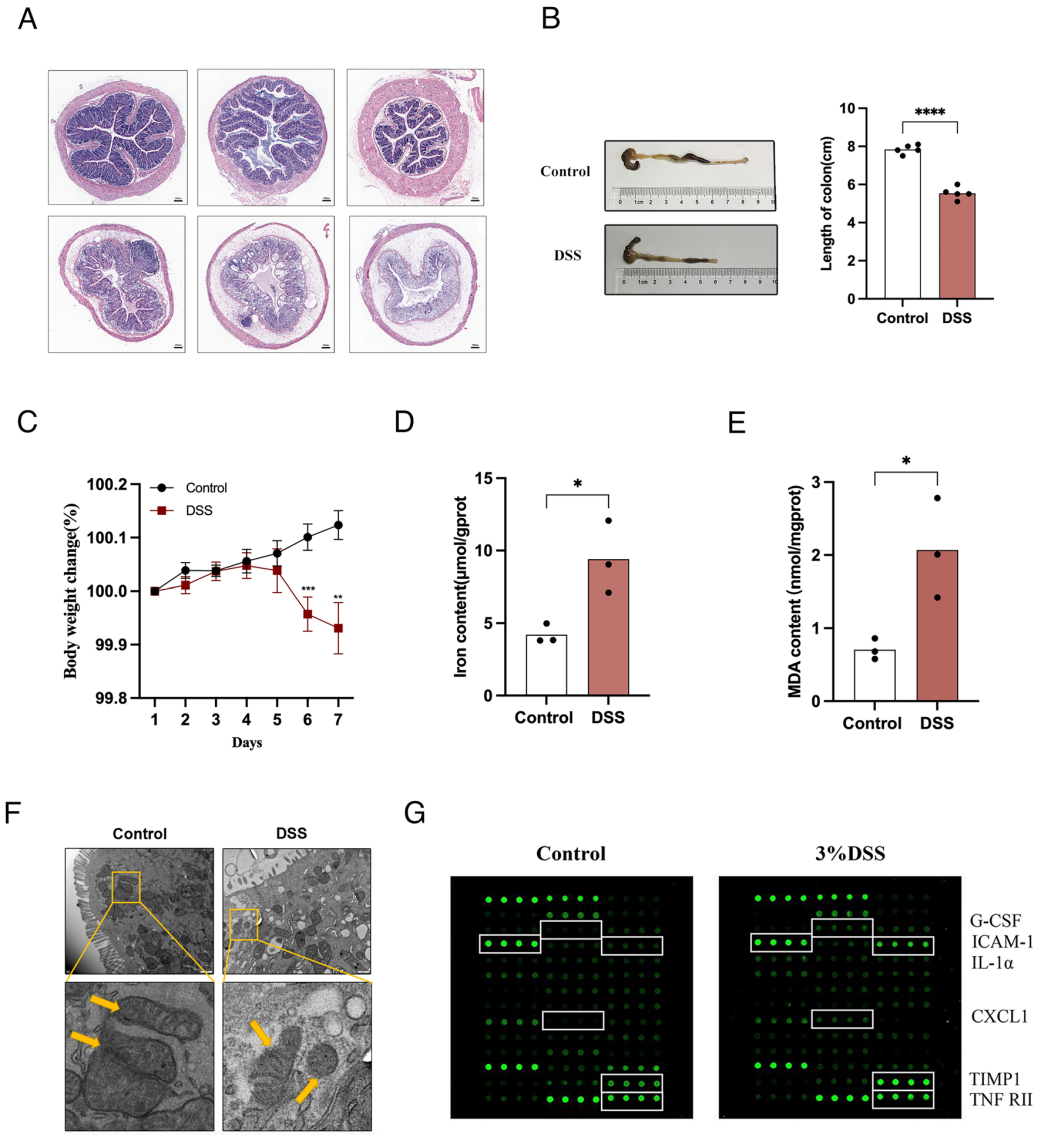
Experimental verification in the UC mouse model **A**, Representative images of colonic mucosal samples from DSS-treated mice and control mice (HE-stain, 20 [scale 50 μm]). Colon length (**B**) and body weight change (**C**) in control and DSS-treated mice. Iron content (**D**), MDA concentration (**E**) and mitochondrial morphology (**F**) in control and DSS-treated mice. (**G**) Inflammatory factor chip analysis of differences in inflammatory factors in mouse intestinal mucositis. **P < 0.01; ***P < 0.001; ****P < 0.0001.

The colitis mouse model was used for in vivo experiments to demonstrate the existence of ferroptosis and neutrophil infiltration. Histopathological examination of DSS-induced colitis mice revealed structural destruction of the colonic mucosa, increased infiltration of inflammatory cells, and destruction of crypts (Fig. 9A). Mice with colitis had significantly shorter colons (Fig. 9B) and significant weight loss (Fig. 9C). We further detected three important iron death indicators. We found that DSS induced iron overload (Fig. 9D) and increased MDA levels (Fig. 9E) in mice. In addition, mitochondrial atrophy and a reduction in the number of mitochondrial cristae in intestinal epithelial cells were observed via transmission electron microscopy (Fig. 9F). We used an inflammatory factor microarray to detect 40 kinds of inflammatory factors in intestinal mucosal tissue specimens (fold change > 1.2, p < 0.05) and found 6 kinds of inflammatory factors related to neutrophil chemotaxis (Fig. 9G). These results indicate the presence of ferroptosis and neutrophil phenotype.

## Discussion

Clinical UC is heterogeneous, as its symptoms range from endoscopic findings to treatment response^45^. The pathophysiological mechanism is complex, and medical interventions are nonspecific, which leads to inconsistent treatment responses^46,47^. This imposes a heavy burden on patients and medical resources, and our previous study showed that Crohn's disease patients suffer many problems, such as lack of disease and drug information and heavy physical and mental burdens^48^. In this study, we performed unsupervised clustering of UC transcriptome data based on ferroptosis and neutrophil gene sets and identified four molecular subtypes with different biological characteristics and levels of immune infiltration. In addition, machine learning was used to screen out four major contributing genes and confirm their diagnostic efficacy and ability to predict drug response. Finally, human and animal samples were used to verify these conclusions.

IBD is a heterogeneous disease with multiple pathogenic factors and multiple pathogeneses and is associated with individual genetic background and immune function. The differences in individual biological traits are the result of interactions between molecules (genes, proteins) that make up different biological individuals. The relationships between these molecules are complex, and the expression of a certain trait is often the result of multiple genes, synergy or antagonism. Our study explored the heterogeneity of UC based on ferroptosis and neutrophil gene sets, ultimately identifying four phenotypes of UC with distinct biological functions. The ferroptosis phenotype may constitute the best group for antiferroptosis therapy to achieve precise protection of the intestinal epithelium. The neutrophil phenotype may be more suitable for immunotherapy. The quiescent phenotype may not be sensitive to either treatment. We were particularly interested in the mixed phenotype because this group had high expression of both iron death-related and neutrophil signature genes. These findings were also confirmed by the results of GSEA and KEGG analyses of this group. Precision therapy is the future direction of IBD treatment, and our study identified four subgroups of UC patients. This stratification may provide directions for precision therapy. However, additional experimental and clinical studies are needed to confirm these findings.

In the past 20 years, great progress has been made in the treatment of IBD. However, but there is a lack of effective markers available for the whole treatment process, and these markers cannot identify the disease early at the molecular level or prevent its development. Our study further screened the importance of genes associated with four molecular subtypes through machine learning and ultimately identified four hub genes (*FTH1, AQP9, STEAP3* and *STEAP4*). In addition, we found that four hub genes have a strong ability to diagnose UC, and ANN analysis revealed that combined diagnosis improved this ability. These results suggest that the four hub genes may be potential biomarkers for UC.

Ferritin heavy chain 1 (*FTH1*) encodes the heavy subunit of ferritin, the main iron storage protein in prokaryotes and eukaryotes^49^. Research has shown that FTH1 is a key protein involved in ferroptosis, and our previous studies have shown this^8^. Aquaporins (AQPs) are a family of water-selective membrane channels. The gene encodes a member of the aquaporin subgroup that is a protein that allows a wide range of uncharged solutes to pass through, and the encoded protein may also play a role in specialized white blood cell functions, such as the immune response and bactericidal activity. A previous study showed that *AQP9* may play a role in regulating tissue-specific physiological properties in tight junctions in UC^50^. In 2005, Ohgami RS et al. first reported that *STEAP3* is involved in iron ion balance^51^. Later, it was found that *STEAP4* has reductase activity, which can promote the reduction of Fe^3+^ to Fe^2+^, and that the reductase activity is stronger than that of *STEAP3*^52,53^. We validated the protein expression of AQP9 and STEAP4 in human samples, and compared to that in normal individuals. The protein expression of AQP9 and STEAP4 was significantly upregulated in patients with UC. Due to the limited sample size, these results do not adequately reflect clinical heterogeneity. It is necessary to confirm the existence of two phenotypes, as this is the basis for establishing molecular subtypes. Therefore, in a mouse model of colitis, we detected the occurrence of ferroptosis, as indicated by iron overload, lipid peroxidation levels, and characteristic mitochondrial structural changes. An inflammatory factor chip also revealed changes in inflammatory factors related to neutrophil chemotaxis. These experimental results all demonstrated the potential role of ferroptosis and neutrophils in UC. However, the animal experiments in this study did not prove heterogeneity. It is necessary to establish different types of animal models based on clinical heterogeneity, which can provide methods for precise clinical treatment.

IFX is a TNF-α-specific binding human and mouse chimeric IgG1 monoclonal antibody and is the first biologic agent approved for the treatment of IBD^54^. It has significant efficacy in inducing remission and maintenance therapy, maintaining fistula closure in IBD patients, preventing recurrence after remission, and reducing surgical risk and hospitalization rate. According to long-term follow-up and clinical trial studies, approximately 37% of patients initially achieve remission with IFX but develop secondary loss of response over time, with a 13% risk of secondary loss of response per year^44,55^. According to our study, *AQP9* and *STEAP4* can predict the treatment response of patients to IFX, and this can assist with the clinical medication decisions for UC patients. However, additional experiments are needed to verify this phenomenon.

However, there are still many limitations in this study. The conclusions obtained from the analysis of the GEO dataset based on bioinformatics and machine learning have not been fully validated by experiments. We carried out human sample and animal experiments to demonstrate the reliability of our conclusions. However, additional molecular experiments are needed to prove this phenomenon. In the future, a large number of clinical samples are still needed for heterogeneity verification. In addition, given the high consistency of model animals, it is still necessary to consider how to replicate the heterogeneity of clinical patients in animal experiments.

In summary, we introduced ferroptosis and neutrophil gene sets into the UC heterogeneity classification system. Four molecular subtypes were identified, and four hub genes were selected by machine learning as diagnostic markers for UC. Among these genes, *AQP9* and *STEAP4* were able to predict the therapeutic response to IFX. Exploring heterogeneity at the molecular level in UC patients can assist in precision treatment, which can reduce the medical burden on patients and prevent the waste of social resources.

### Supplementary Information

Supplementary Figure 1.

Supplementary Information 2.

Supplementary Information 3.

## Data Availability

All data are available from the first author (sunshaopeng0903@qq.com).
