# Supplementary material for: Biological characteristics of molecular subtypes of ulcerative colitis characterized by ferroptosis and neutrophil infiltration
Source: Sci Rep. 2024 Apr 25;14:9510. doi: 10.1038/s41598-024-60137-z (PMC11045816; doi:10.1038/s41598-024-60137-z)
Supplement: Supplementary file 1 — Supplementary Figure 1. [file 41598_2024_60137_MOESM1_ESM.pdf]

Supplementary Fig 1: The full-length gels and blots for Fig.8D

WB experimental procedure

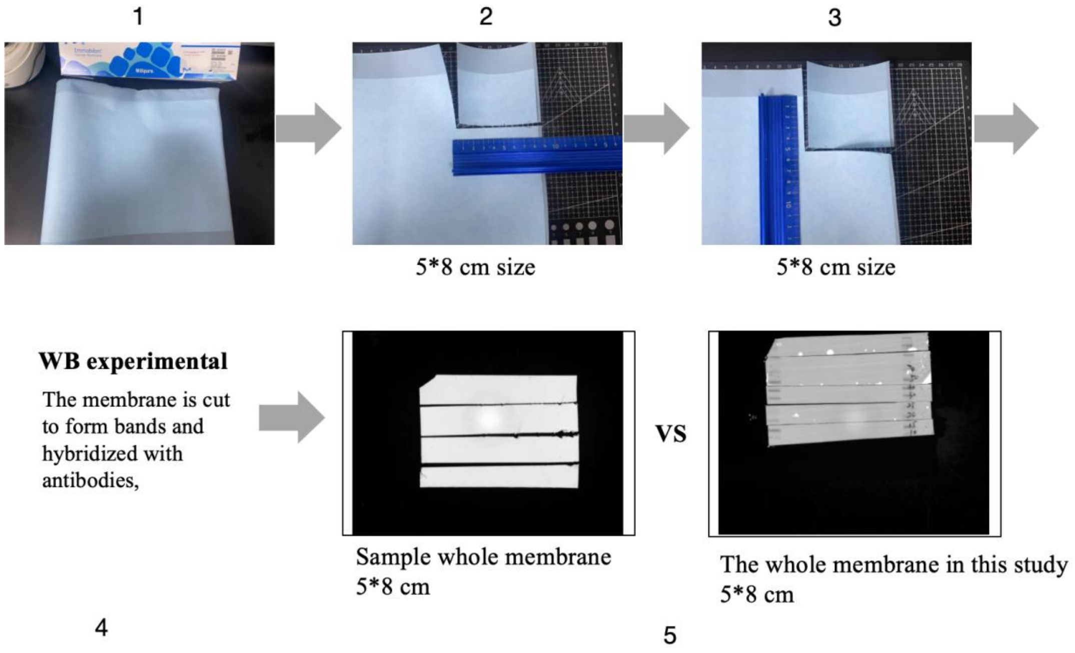

First of all, a complete membrane for WB experiment was cut by hand (the membrane in this experiment was 5*8cm in size).[step1-3]

Second, after the sample protein is transferred to the membrane, we cut the membrane laterally according to the position indicated by the maker protein, forming bands and hybridizing the antibodies.[step4]

Therefore, the edge of the whole membrane provided in this experiment has cut marks, but it is still complete. Observe [step 5] to prove that the complete film in the example is the same size as the film in the experiment, indicating that the film provided in the experiment is also complete.

The gels and blots for Fig.8D

STEAP4

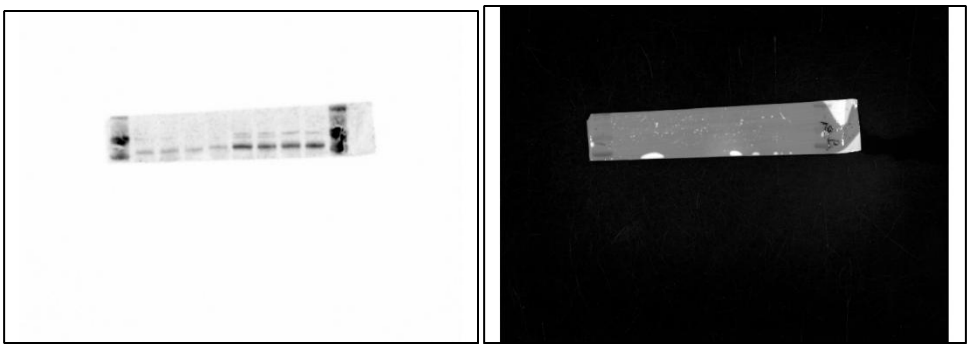

AQP9

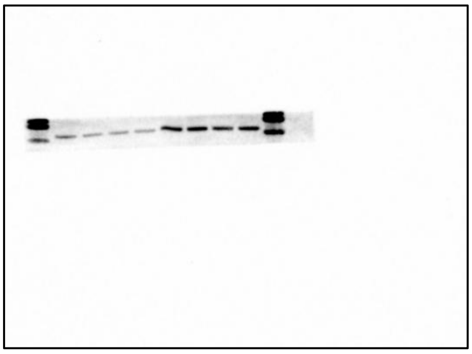
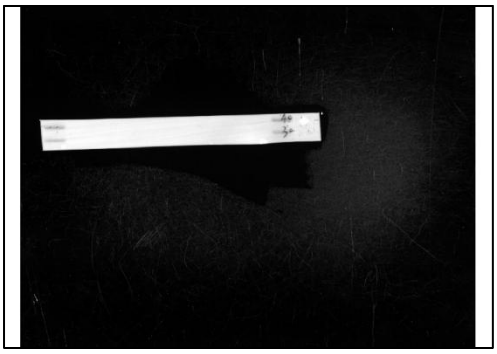

GAPDH

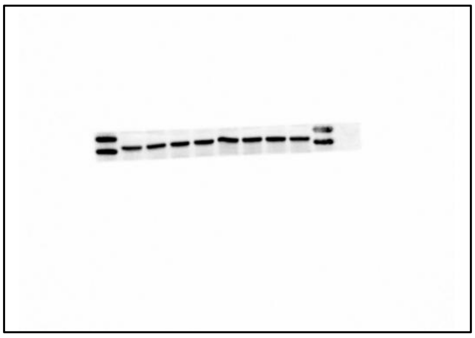
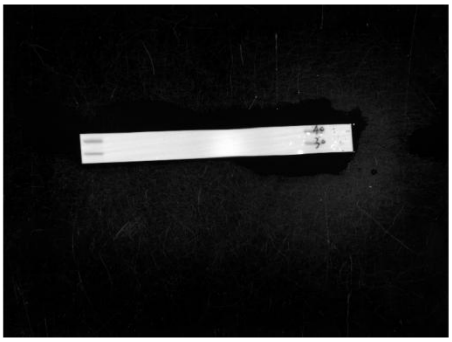

Whole membranes

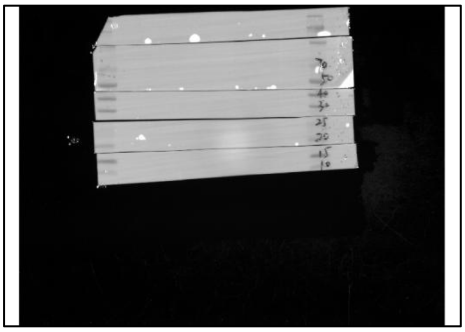

The authors of this paper are responsible for the authenticity of WB experiment results.
