# Supplementary material for: Biological characteristics of molecular subtypes of ulcerative colitis characterized by ferroptosis and neutrophil infiltration
Source: Sci Rep. 2024 Apr 25;14:9510. doi: 10.1038/s41598-024-60137-z (PMC11045816; doi:10.1038/s41598-024-60137-z)
Supplement: Supplementary file 3 — Supplementary Information 3. [file 41598_2024_60137_MOESM3_ESM.docx]

**To help the reader evaluate the principle, we provide more detailed results of machine learning in this study.**

**The Boruta algorithm** is a wrapper feature selection method that compares the importance of the original feature and the randomly generated shadow feature. A feature that is significantly better than a shadow feature is labelled an “important feature”


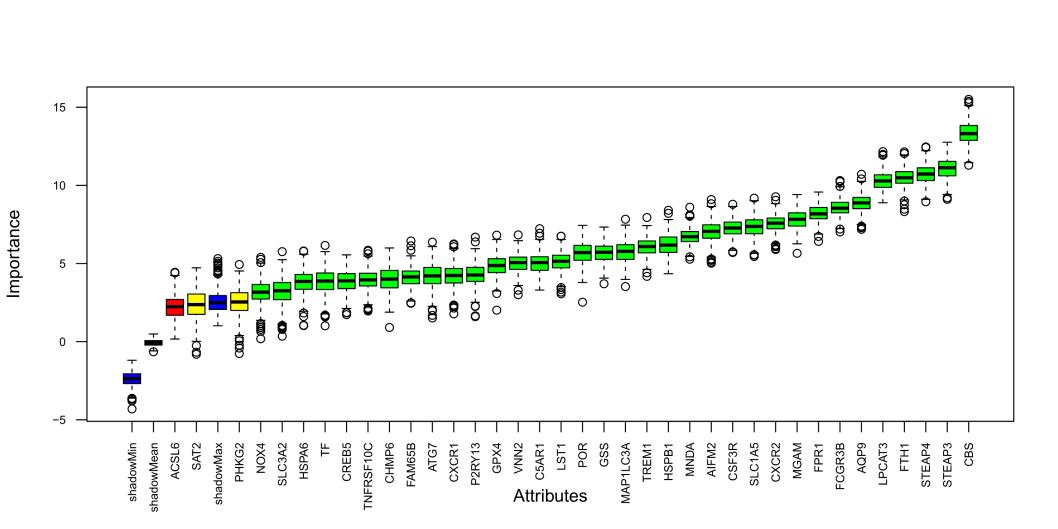


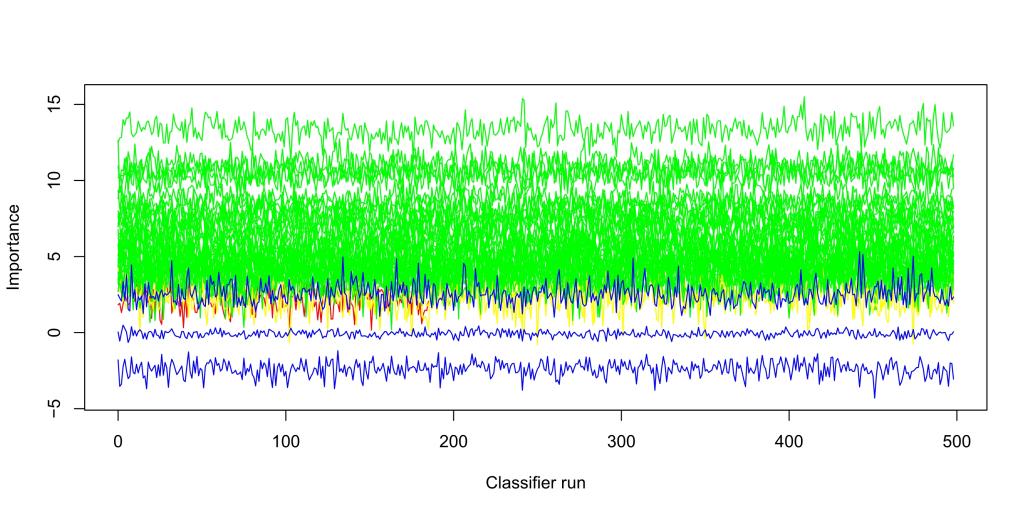


**Least absolute shrinkage and selection operator (LASSO)** regressioncan reduce the impact of multicollinearity on regression results by reducing the coefficient of the relevant independent variables to 0 through the correlation between them.


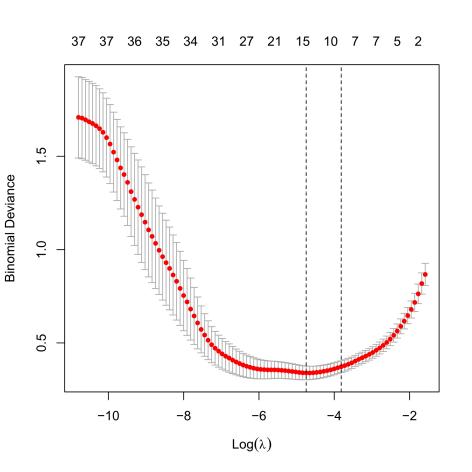

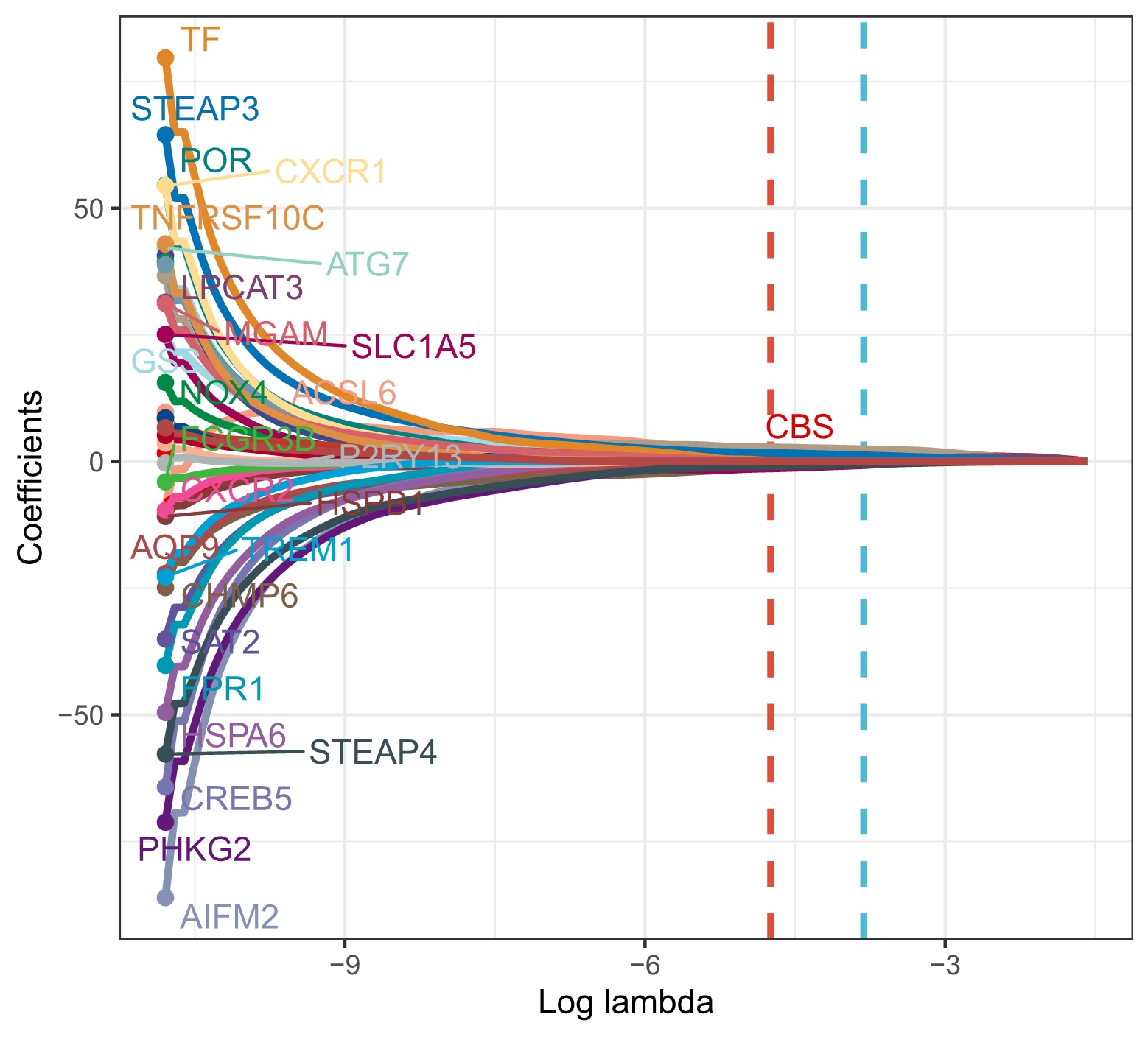


**Support vector machines (SVMs)** are supervised learning algorithms used to solve binary classification problems. This method can find an interface so that all samples are correctly divided into two categories, and the distance between the samples and the interface is maximized


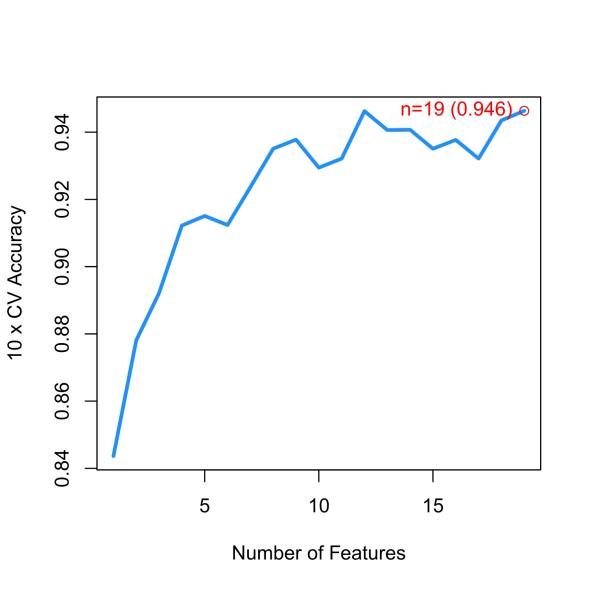

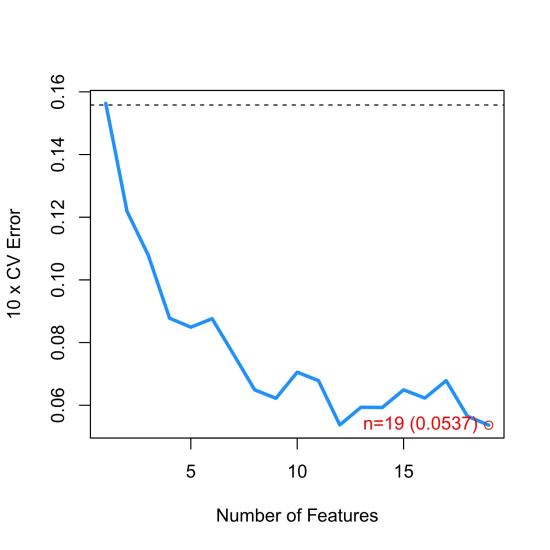


**RandomForest** is a classifier that contains multiple decision trees, and the category of its output is determined by the mode of the categories output by the individual trees.


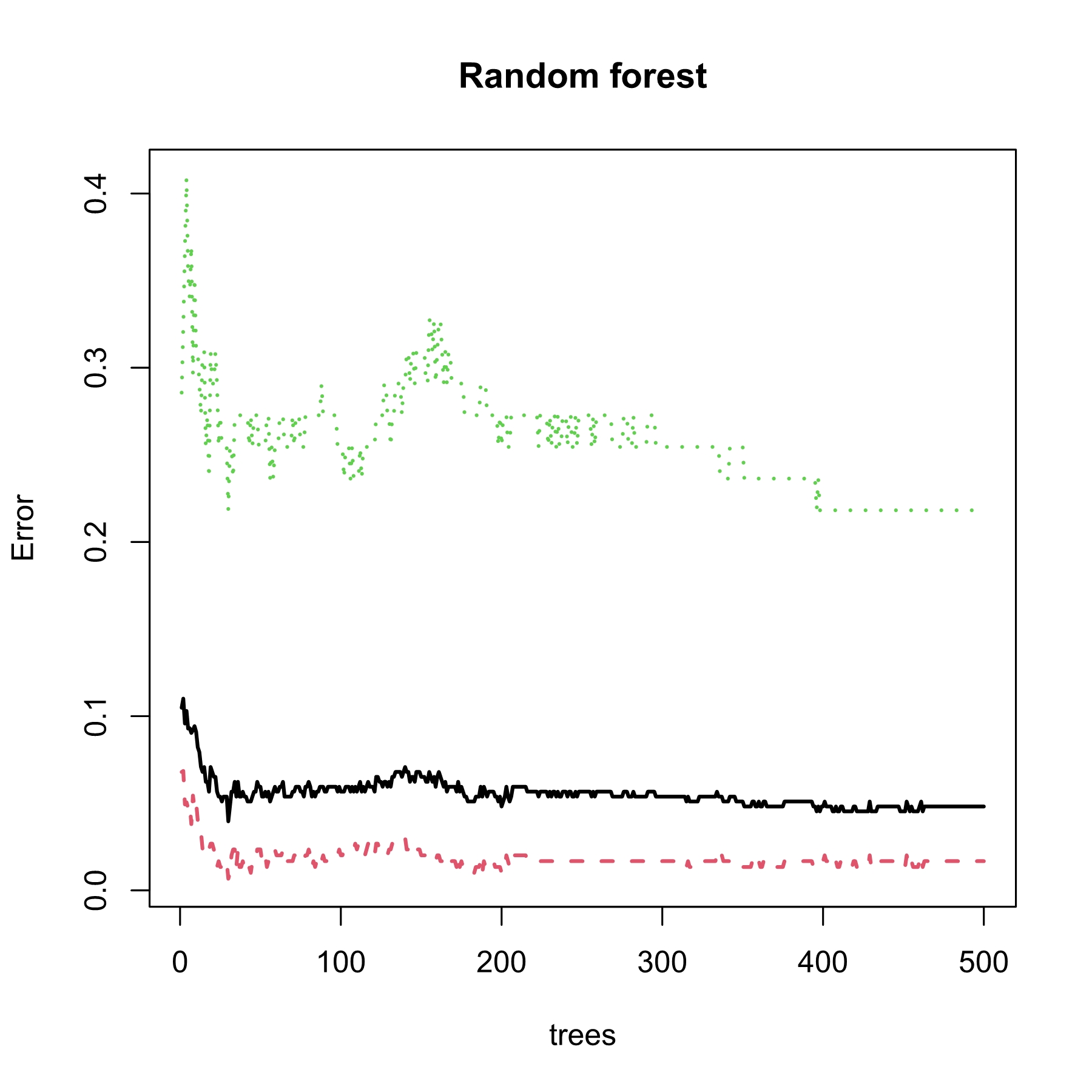

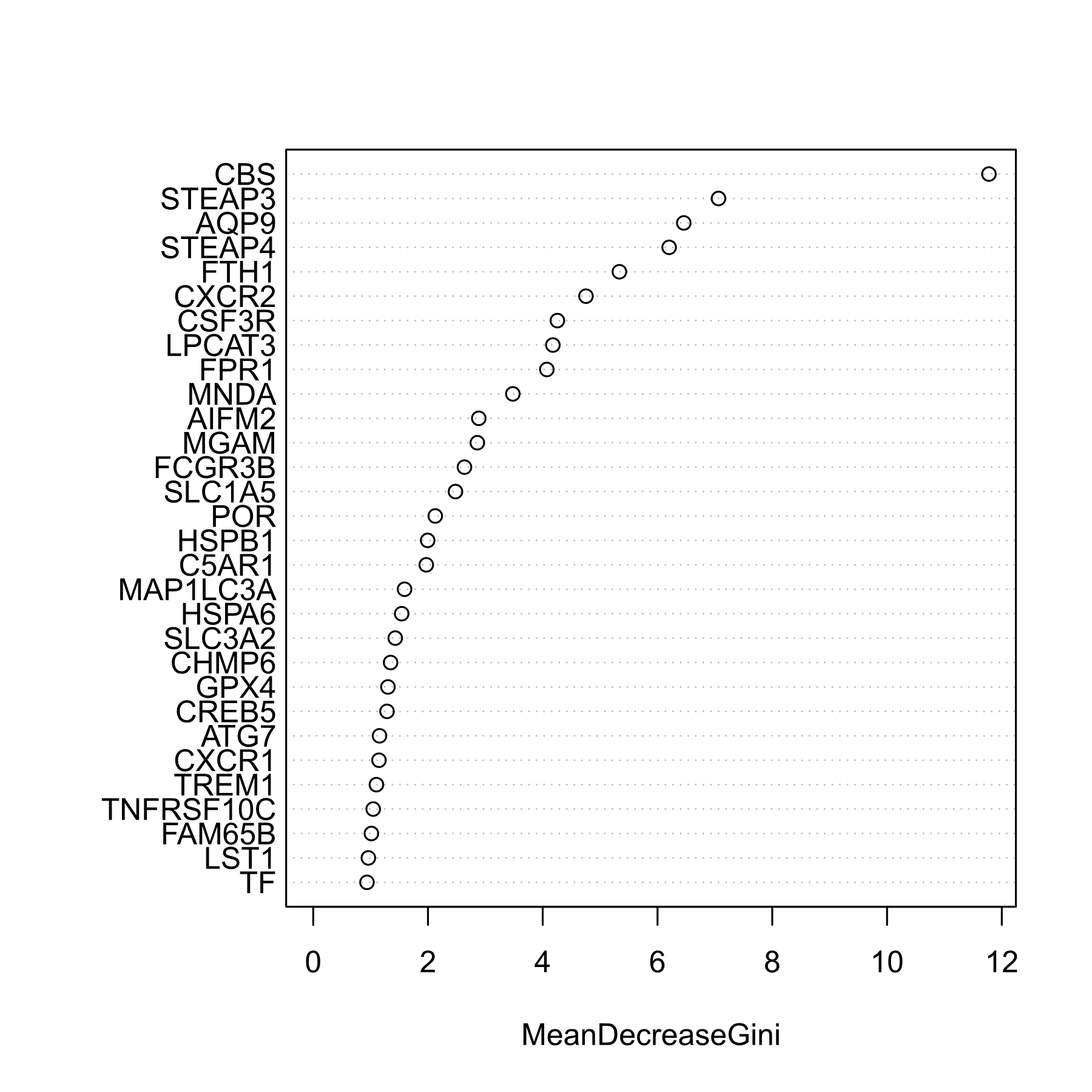


Extreme gradient boosting **(XGBoost)** is a boosting algorithm that can integrate many weak classifiers to form a strong classifier


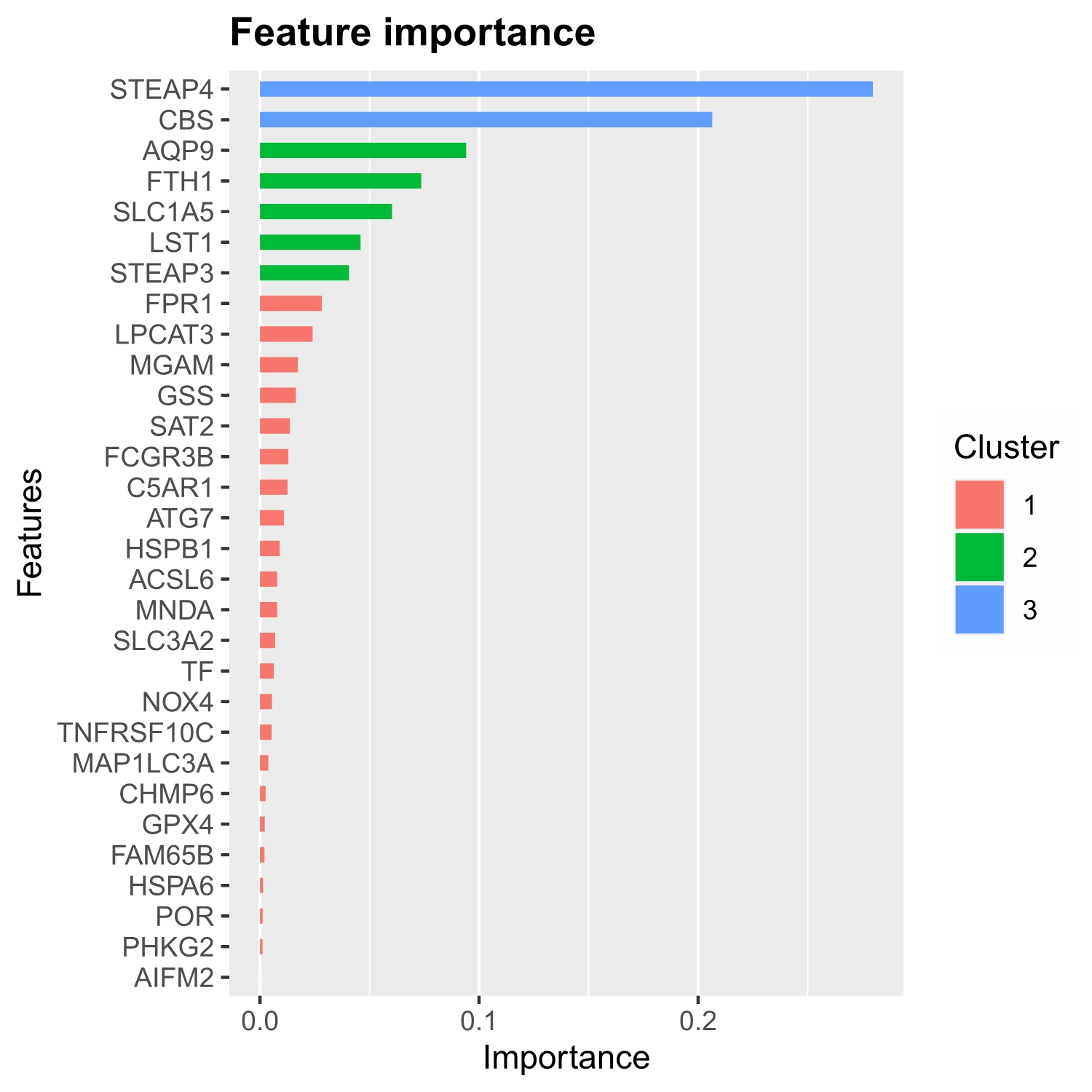


**We consider the overfitting possibilities of machine learning.** For this reason, we did the following two things at the beginning of the design.

(1) Expanding the sample size. We combined multiple data sets to complicate the distribution characteristics of the data and avoid the bias caused by a single data set. (2) Multiple machine learning is used to identify diagnostic genes, and the intersection is finally taken. If multiple algorithms agree that a certain gene is important, then we believe the result is robust, so as to avoid the deviation caused by a single algorithm. For example, lasso is a regularization method for linear regression and feature selection. Its basic principle is to introduce L1 regularization term into the loss function, and realize sparse model parameters and feature selection by minimizing the data fitting error and the sum of regularization term, which can reduce overfitting to a certain extent.
